# 274. Potential HIV vaccine gp41 epitope targeting antibodies identify peptides with similarity to proposed Kawasaki disease related peptide, suggesting non-specific mimotope targeting of acidic amino acid enriched regions

**DOI:** 10.1093/ofid/ofad500.346

**Published:** 2023-11-27

**Authors:** Sojar Hakimuddin, Sarah Baron, Mark D Hicar

**Affiliations:** University at Buffalo, buffalo, New York; University at Buffalo, buffalo, New York; University at Buffalo, buffalo, New York

## Abstract

**Background:**

We have previously isolated a highly mutated (83% homologous to predicted heavy chain germline) antibody (Ab) termed C group 76-Q13-6F5 (6F5) that targets a conformational epitope on gp41. 6F5, though non-neutralizing, has the capacity to mediate Ab dependent cell cytotoxicity (ADCC). When the variable chain (predicted to be VH1-02 derived) was mutated to germline (termed C group 76 ancestor, or 76Canc), surprisingly this Ab still exhibited significant ADCC activity. Many HIV vaccine strategies are focused on raising highly mutated Abs. We propose that there would be an advantage to developing vaccines related to epitopes that permit functional targeting by Abs using germline variable gene sequences.

**Methods:**

To explore potential protein targets for vaccination strategies to raise and develop such Abs, we interrogated a peptide array of 29,127 linear peptides using PEPperCHIP® Human Epitome Microarray. We then confirmed peptide binding by Western blot and ELISAs. We also assessed binding to CDI laboratories HuProt protein microarray, containing > 21,000 human proteins.

**Results:**

76Canc specifically recognized a number of peptides enriched for glutamic and aspartic acid residues (top hit DEEEEYDEDEYEYDE). Meme analysis of positive peptides revealed a peptide sequence most similar to Hepatitis C virus, similar to a peptide implicated in Kawasaki disease (KD). We confirmed specific binding of four of the top peptide hits, including hepatitis C peptide recognition. We then confirmed binding of 76Canc-related Abs to a published optimized KD related peptide (KPAVIPDREALYQDIDEMEEC). Serum from KD and infectious controls was used to compete with biotinylated 76Canc-related Abs. Serum Abs targeting this epitope showed no specific correlation to having KD. Autoantigen screening of 76Canc identified a single human protein of interest that did contain acidic amino acid rich regions.

**Figure 1:**
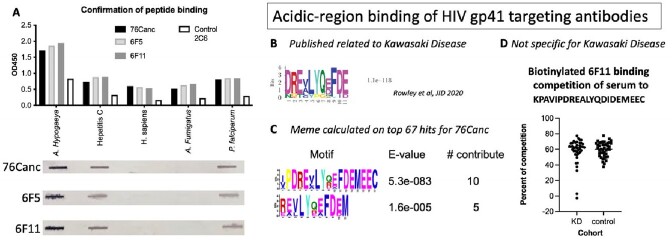
HIV-1 gp41 antibodies recognize peptides similar to peptide implicated in Kawasaki Disease

**Conclusion:**

This study reveals acidic motif targeting by specific anti-gp41 Abs and the derived germline Ab, but no evidence that these Abs are related to inflammation similar to KD. Cautious development of targeting such Abs by vaccination is warranted. Future structural comparison of these peptides with native proteins and binding competition studies are needed to confirm mimotope binding.

**Disclosures:**

**Mark D. Hicar, MD/PhD**, Pfizer: site investigator for 2 trial

